# Variational Temporal Deconfounder for Individualized Treatment Effect Estimation with Longitudinal Observational Data

**DOI:** 10.21203/rs.3.rs-2536079/v1

**Published:** 2023-02-06

**Authors:** Zheng Feng, Mattia Prosperi, Yi Guo, Jiang Bian

**Affiliations:** University of Florida; University of Florida; University of Florida; University of Florida

**Keywords:** Causal inference, Individualized treatment effects, Observational data, Interpretable AI

## Abstract

**Purpose:**

This paper proposes a new approach, Variational Temporal Deconfounder (VTD), for estimating individualized treatment effects (ITE) from longitudinal observational data, where we address the hidden confounding issues by using proxies (i.e., surrogate variables that serve for unobservable variables).

**Methods:**

We build VTD by incorporating a variational recurrent autoencoder that learns the latent encodings of hidden confounders from observed proxies and an ITE estimation network that takes the learned hidden encodings to predict the probability of receiving treatments and potential outcomes.

**Results:**

We test VTD on both synthetic and real-world clinical data, and the results from synthetic data experiments demonstrate VTD’s effectiveness in deconfounding by outperforming existing methods, while results from two real-world datasets (i.e., Medical Information Mart for Intensive Care version III [MIMIC-III] and the National Alzheimer’s Coordinating Center [NACC] database) suggest that the performance of the VTD model outperforms existing baseline models, however, varies depending on the assumptions of underlying causal structures and availability of proxies for hidden confounders.

**Conclusion:**

The VTD offers a unique solution to address the confounding bias without the “unconfoundedness” assumption when estimating the ITE from longitudinal observational data. The elimination of the requirement for the “unconfoundedness” assumption makes the VTD more versatile and practical in real-world clinical applications of personalized medicine.

## 1 Introduction

Estimating treatment effect—the causal effect of a given treatment or intervention on an outcome, plays an important role in evidence-based medicine, providing quantified measurements of benefits or harms for the treatment of interest, which help regulators to make regulatory decisions, health care community to develop guidelines and decision support tools, and clinical professionals to decide the treatment choices in their clinical practice. Randomized controlled trials (RCTs) have been widely used as the gold-standard to estimate the average treatment effects (ATE), measuring the difference in average outcomes between individuals in the treatment group and those in the control group. In a well-designed RCT, patients are randomly assigned to the control and treatment groups, such that the units in the treatment vs. control groups are identical across all known and unknown factors to reduce the potential bias [1]. However, there are limitations of RCTs as they are not only time-consuming and logistically complex to conduct, but also the study results may not generalize beyond the study population, especially to the real-world populations where the treatment will be applied [2]. In recent years, the rapid growth of electronic health record (EHR) systems has made large collections of longitudinal observational real-world data (RWD) available for research to generate real-world evidence (RWE) [3]. The U.S. Food and Drug Administration (FDA) provided guidance on using RWD like EHRs and claims data to support regulatory decision-making [4] and has recently approved a new use for Prograf (tacrolimus)—originally approved to prevent organ rejections in liver transplants, and now approved for lung transplants, based on a observational study providing RWE of effectiveness [5]. Nevertheless, the observational, non-interventional study needs to be well-designed, accounting for the inherent biases in observational data such as confounding issues and selection biases. Further, moving beyond ATE, there is also a strong desire to obtain an individualized treatment effect (ITE), considering the heterogeneity of the target patient population and their differential responses to the same treatment. In recent years, ITE estimation based on more accessible observational data like EHRs has been a thriving research area to fill the gap [6].

One of the most critical issues of estimating ITEs from observational data is confounding - when variables can affect both the outcomes and the interventions [7]. These variables are thus called confounders. For example, socioeconomic status can affect both the medication a patient has access to and the patient’s general health. Therefore, socioeconomic status acts as a confounder between medication and health outcomes. If confounders can be measured, the most common way to counter their effect is by “controlling” them in the models [8]. Many approaches for estimating ITE from observational data have been proposed according to this solution, which can be categorized into 2 groups: (1) covariate adjustment [9–12], and (2) propensity score re-weighting [13–15]. Most of these approaches are built on a commonly used assumption of “unconfoundedness,” where all variables that affect both the interventions and outcomes are observed and measured. However, the unconfoundedness-based models will lead to biased ITE estimation when certain confounders are hidden or unmeasured [16]. In reality, it is unlikely that we can observe and/or directly measure all confounders in real-world observational studies. For example, RWD like EHRs often do not have variables such as environmental factors or personal preferences, which are potential confounders. A possible way of modeling hidden confounders is through modeling their proxies (i.e., surrogate measures). For example, stigma is an important factor in clinical care and outcomes for a variety of conditions, from infectious diseases to mental health. However, a stigma questionnaire might not be always administered to people during healthcare encounters. Mental health assessment and other measurements, however, might be available and be used as surrogates [17].

Several approaches have built deconfounding ITE estimation models by using proxy variables. The multiplicity of causes [18] and matrix factorization [19] are used to infer the confounders from missing or proxy variables. More recently, the variational autoencoder (VAE) [20]—a deep generative model with powerful hidden representation learning ability, has been applied to model hidden confounders [8, 21, 22] and achieved superior performance. However, these variational generative model-based approaches are designed for cross-sectional settings and cannot be directly adopted for a longitudinal setting. In real-world clinical practice, EHRs contain rich time-dependent patient information such as lab results, vital signs, and medication use across their encounters with the health system. With such longitudinal data, we can answer some essential questions such as what is the optimal time to administer a treatment, when the treatment regime needs to be stopped, or in which order treatments should be given to obtain the best treatment response [6]. Only a few attempts have built longitudinal ITE models [23, 24], and none of them have tried to use the variational generative approach to model the hidden confounders over time.

In this paper, we propose the Variational Temporal Deconfounder (VTD), a novel method for ITE estimation that leverages the variational autoencoder to model hidden confounders in a longitudinal setting. Instead of assuming no unobserved hidden confounders, we create embeddings of latent variables to recover the distributions of hidden confounders from the proxies over the observational data space. Our approach is two-fold: (1) a transformer-based factor model that can infer the latent random variables with a variational autoencoder that learns the hidden confounders from variations of observed proxies while capturing the dependencies among the hidden confounders at neighboring timesteps; and (2) a timestep-wise variational lower bound together with the prediction loss to integrate joint training of the latent factor model with the ITE estimation task. We highlight our VTD, same as the time series deconfounder [23], works as an unbiased ITE estimation approach requiring weaker assumptions than standard methods over observational data. To show the effectiveness of VTD, we first conducted a simulation study to investigate VTD’s capability to infer latent variables where we explicitly created hidden confounders. Then we evaluate VTD on two real-world datasets: (1) the Medical Information Mart for Intensive Care III (MIMIC-III) dataset with patients admitted into Intensive Care Units (ICU), and (2) data from with the National Alzheimer’s Coordinating Center (NACC) database to evaluate our proposed methods.

## 2 Methods

### 2.1 Problem formulation

**Figure 1:**
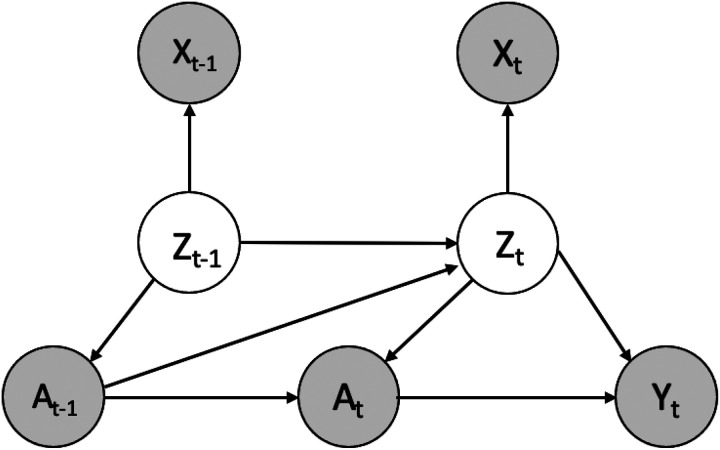
Graphical factor model of the proposed Variational Temporal Deconfounder (VTD).

Let random variables ${\text{X}}_{t}^{\left(i\right)}=\left[x_{t,1}^{\left(i\right)},x_{t,2}^{\left(i\right)},\ldots ,x_{t,p}^{\left(i\right)}\right]\in {𝒳}_{t}$ denote observed p dimensional time-dependent covariates of a patient (i) with time-stamp *t* = 1, … , *T* and *i* = 1, … , *N*. Let random variables ${\text{A}}^{\left(i\right)}=\left[a_{1}^{\left(i\right)},a_{2}^{\left(i\right)},\ldots ,a_{t}^{\left(i\right)}\right]\in {\text{a}}_{t}$ denote a binary treatment assignment, and ${\text{Y}}^{\left(i\right)}=\left[y_{1}^{\left(i\right)},y_{2}^{\left(i\right)},\ldots ,y_{t}^{\left(i\right)}\right]\in {𝒴}_{t}$ be observed outcomes over time-stamp t. For simplicity, we include static features as part of observed covariates **X** since it does not change our assumptions. For patient i = 1, … , N, and across time stamp t = 1, … , T, we denote an observed dataset as $𝒟={\left({\left\{{\text{x}}_{t}^{\left(i\right)},{\text{a}}_{t}^{\left(i\right)},{\text{y}}_{t}^{\left(i\right)}\right\}}_{t=1}^{T}\right)}_{i=1}^{N}$. We emphasize that observed covariates ${\text{x}}_{t}^{\left(i\right)}$ in 𝒟 are proxies of hidden confounders. We denote the unobserved confounders for proxies ${\text{x}}_{t}^{\left(i\right)}$ as r-dimensional random variables **Z** where ${\text{Z}}_{t}^{\left(i\right)}=\left[Z_{t,1}^{\left(i\right)},Z_{t,2}^{\left(i\right)},\ldots ,Z_{t,r}^{\left(i\right)}\right]\in {𝒵}_{t}$. Figure 1 shows the causal structure between hidden confounders **Z** and other variables. ]

We adopt the potential outcome framework under the longitudinal setting proposed by Robins and Hernán [25] who extended it from the static setting of Neyman [26] and Rubin [27]. Let ${\bar{\left(\cdot \right)}}_{t}$ denote the historical covariates collected before time t. For each patient, given observed covariates $\bar{\text{X}}=\left[x_{1},x_{2},\ldots ,x_{t}\right]\in {\bar{x}}_{t}$ and treatment of $\bar{\text{A}}=\left[a_{1},a_{2},\ldots ,a_{t}\right]\in {\bar{a}}_{t}$,we want to estimate individualized treatment effects (ITE), i.e., potential outcomes ${\text{Y}}_{\left(\bar{a}\right)}$ conditioned on ${\bar{\text{A}}}_{t-1}$, ${\bar{\text{X}}}_{t}$ as

1 $$E\left[{\text{Y}}_{\left(\bar{a}\geq t\right)}|{\bar{\text{A}}}_{t-1},{\bar{\text{X}}}_{t}\right]$$

We adopt two standard assumptions [28] for ITE estimation:

#### Assumption 1

Consistency. If, then the potential outcome for treatment assignment is the same as the observed outcome, i. e.,.

#### Assumption 2

Positivity (Overlap). If then

Other than these two assumptions, the majority of other ITE methods also assume unconfoundedness or sequential ignorability, i. e.,

2 $${\text{Y}}_{t+1}\left[{\text{a}}_{t}\right]\;{\text{A}}_{t}|{\bar{\text{A}}}_{t-1},{\bar{\text{X}}}_{t}$$

for all **a**_*t*_ ∈ **𝒜**_*t*_ and *t* ∈ {0, … , T}, which holds only if there are no hidden confounders. In our setting, we observe proxies ${\bar{\text{X}}}_{t}$ instead of hidden confounders ${\bar{\text{Z}}}_{t}$, where unconfoundedness is violated, and using standard methods will result in biased ITE estimation.

We address this by using the VTD, which learns a hidden embedding that reflects the true hidden confounders **Z**_*t*_ from variations of observed proxies **X**_*t*_ and also captures the dependencies among **Z**_*t*_ at neighboring timesteps.

### 2.2 The Variational Temporal Deconfounder model

We introduce our VTD model as follows: (1) the architecture of the VTD that consists of a variational recurrent autoencoder and an ITE block to produce the hidden embedding and ITE estimation, respectively; and (2) the variational bound of VTD, which ensures the embedding of the hidden confounders can be learned by standard gradient-based optimization.

#### 2.2.1 The architecture of the VTD

**Figure 2:**
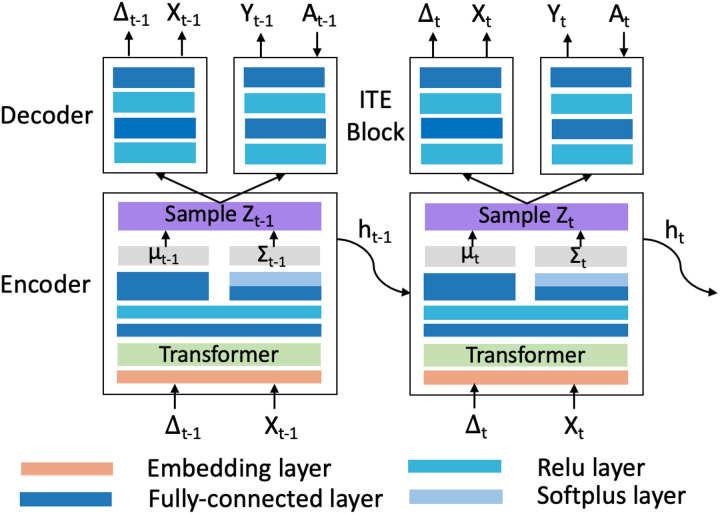
The proposed Variational Temporal Deconfounder (VTD) model architecture.

The VTD consists of two main components: (1) a variational recurrent autoencoder, which learns the latent variables of hidden confounders **z**_*t*_ the observed proxies x_t_; and (2) an ITE estimation block, which takes learned hidden embedding ${\overset{\prime }{\text{z}}}_{\text{t}}$ to predict the probability of receiving treatment ${\overset{\prime }{\text{a}}}_{t}$ and potential outcome $\overset{\prime }{{\text{y}}_{t}}$ . We illustrate the architecture of VTD in Fig. 2.

##### (1) The variational recurrent autoencoder.

The variational recurrent autoencoder uses a recurrent encoder-decoder framework where a transformer is introduced to adjust **z**_*t*_ for the time-varying structure of a longitudinal setting shown in Fig. 1. The encoder maps the inputz proxies **x**_*t*_ from the observed space to the latent space of hidden confounders z_t_. In the encoder, a transformer [29] takes a sequence of observed proxies x and outputs the hidden states h accordingly, followed by a fully-connected layer *ϕ*_*enc*_ that takes the outputs of the transformer layer and maps the hidden states **h**_*t*_ and **h**_*t*−1_ of each time step **t** onto the latent embedding ${\overset{\prime }{\text{z}}}_{t}$, i.e.,

3 $$\overset{\prime }{{\text{z}}_{t}}=\phi_{enc}\left({\text{h}}_{t-1},{\text{h}}_{t}\right)$$

Further, when implementing the transformer, we also consider the elapsed time between the patient’s two consecutive encounters (with the health system) in order to take into account the time-varying effect of clinical events (e.g., a bone fracture happened a year ago would have a different effect on the patient’s current health status comparing to a bone fracture happened a week ago). Thus, we add the elapsed time ***Δ****t* along with the input **x** and define the generation of the hidden state **h**_*t*_ as

4 $${\text{h}}_{t}={\text{h}}_{t-1}*ℋ(\Delta t)$$

Then the decoder takes the embedding ${\overset{\prime }{\text{z}}}_{t}$ as input and generates the proxies **x**_*t*_ with a variational mapping function *g*, which takes mapping from ${\overset{\prime }{\text{z}}}_{t}$ and **h**_*t*−1_ to parameters vectors *μ* and *Σ*, and then generate samples as the input of the decoder network*ϕ*_*dec*_

5 $$\overset{\prime }{{\text{x}}_{t}}=\phi_{dec}\left(g\left(\overset{\prime }{{\text{z}}_{t}}\right)\right)$$

##### (2) The ITE estimation block.

Leveraging the learned hidden embedding ${\overset{\prime }{\text{z}}}_{t}$ as the representation of hidden confounders, we estimate the ITE by incorporating two tasks of predicting (i). the probability of receiving treatment ${\overset{\prime }{\text{w}}}_{t}$ and (ii). the outcome ${\overset{\prime }{\text{y}}}_{t}$.

We use a fully-connected layer *f*_*a*_ that takes the embedding ${\overset{\prime }{\text{z}}}_{t}$ to predict the predicted probability of receiving treatment as ${\overset{\prime }{\text{a}}}_{t}$, i.e.,

6 $$\overset{\prime }{{\text{a}}_{t}},=f_{a}\left(\overset{\prime }{{\text{z}}_{t}}\right)$$

We also use a fully-connected layer *f*_*y*_ to predict the outcome ${\overset{\prime }{\text{y}}}_{t}$, which takes the hidden embedding ${\overset{\prime }{\text{z}}}_{t}$ together with the assigned treatments **a**_*t*,_ i. e.,

7 $${\overset{\prime }{\text{y}}}_{t,{\text{a}}_{t}}=f_{y}\left(\overset{\prime }{{\text{z}}_{t}},{\text{a}}_{t}\right)$$

Then we compute the weights using the inverse probability of treatment weighting (IPTW) and extend them to a dynamic setting as follows,

(8) $$\overset{\prime }{{\text{w}}_{t}}=\frac{\text{Pr}(A)}{\overset{\prime }{{\text{a}}_{t}}}+\frac{\left(1-\text{Pr}(A)\right)}{\left(1-\overset{\prime }{{\text{a}}_{t}}\right)}$$

where Pr (*A*) denotes the probability of being in the treated group. By incorporating (7) with our outcome prediction, we define the supervised loss **L**_*s*_ as

(9) $${\text{L}}_{s}=E\left[\overset{\prime }{{\text{w}}_{t}}\left(\overset{\prime }{{\text{y}}_{t}}-{\text{y}}_{t}\right)\right]$$

#### 2.2.2 The variational bound of VTD

VAE was proposed to model complex multimodal distributions of hidden factors over the space of the observed dataset. We define the joint distribution of observed proxies **x** and latent confounders **z** over ***T*** time steps as follows,

(10) $$p\left(x_{\leq T},z_{\leq T}\right)=\prod_{t=1}^{T}p\left(x_{t}|z_{\leq t},x_{<t}\right)p\left(z_{t}\right)$$

In the standard VAE, the latent random variable *z* follows a standard Gaussian distribution. To reflect the causal structure in Fig. 1, we assume *z*_*t*_ follows a a prior Gaussian distribution as

(11) $$z_{t}\sim 𝒩\left(\mu_{t},\Sigma_{t}\right),\text{ where }\left[\mu_{t},\Sigma_{t}\right]=f\left({\text{h}}_{t},{\text{h}}_{t-1}\right)$$

where *f* is a function that maps the hidden states **h**_*t*−1_ and **h**_*t*_ to the parameter space of *μ*_*t*_ and *Σ*_*t*_. And we alsof assume*x*_*t*_ | *z*_*t*_ follows a Gaussian distribution

(12) $$x_{t}|z_{t}\sim 𝒩\left(\mu_{t},\Sigma_{t}\right),\text{ where }\left[\mu_{t},\Sigma_{t}\right]=g\left({\text{z}}_{t}\right)$$

Now our goal is to infer the parameter of the posterior *p* (*z*_≤*T*_|*x*_≤*T*_). By following the paradigm in [30, 31] we introduce the variational distribution *q* (*z*_*t*_ | *x*_≤*t*_, *z*_<*t*_) and transfer the problem of inferencing *p* (*z*_≤*T*_|*x*_≤*T*_) to maximize

(13) $${\text{L}}_{\text{ELBO}}=E_{q\left(z_{\leq t},x_{<t}\right)}\left[\sum_{t=1}^{T}\left(\text{log}p\left(x_{t}|z_{\leq t},x_{<t}\right)-KL\left(q\left(z_{t}|x_{\leq t},z_{<t}\right)||p\left(z_{t}|x_{<t},z_{<t}\right)\right)\right)\right]$$

where **L**_*ELBO*_ denotes the marginal likelihood lower bound (ELBO) of the full dataset. We incorporate the supervised loss of ITE estimation **L**_*s*_ and **L**_*ELBO*_ to define loss **L** as

(14) $$\text{L}={\text{L}}_{s}-\alpha {\text{L}}_{\text{ELBO}}$$

## 3 Experiments

We demonstrate the effectiveness of the VTD in experiments using a synthetic dataset, the MIMIC-III dataset, and the NACC dataset. We show that the VTD reduces confounding bias in ITE estimation from the empirical observation from both experiments. We compared VTD with the following causal inference approaches:

1. **G-formula**, a generalized approach to the standard regression model over the longitudinal setting that can be used to adjust for time-varying confounders [32];
2. **Deep Sequential Weighting (DSW)**, which infers the hidden confounders by incorporating the current treatment assignments and historical information using a deep recurrent weighting neural network [24];
3. **Time Series Deconfounder (TSD)**, which leverages the assignment of multiple treatments over time to enable the estimation of treatment effects in the presence of multi-cause hidden confounders [23].

We report the Rooted Mean Square Error (RMSE) between predicted and ground truth outcomes to measure models’ performance on conventional prediction tasks. To evaluate ITE estimation, the most common measurement is the Precision in Estimation of Heterogenous Effect (PEHE) [33], defined as the mean squared error between the ground truth and estimated ITE, i.e.,

15 $$\text{PEHE}=\frac{1}{N}\sum_{i=1}^{N}{\left(\left(y_{1}^{\left(i\right)}-y_{0}^{\left(i\right)}\right)-\left({\overset{\prime }{y}}_{1}^{\left(i\right)}-{\overset{\prime }{y}}_{0}^{\left(i\right)}\right)\right)}^{2}$$

However, in real-world datasets, the counterfactual is never observed; thus, we use the influence function - PEHE (IF-PEHE) that approximates the true PEHE by “derivatives” of the PEHE function [34].

### 3.1 Datasets

#### 3.3.1 The synthetic data

In the problem formulation section above, we introduced that the treatment assignments $a_{t}^{\left(i\right)}$ at each time step *t* are determined by confounders $q_{t}^{\left(i\right)}$, which also include previous hidden confounders $z_{t-1}^{\left(i\right)}$, current time-varying covariates $x_{t}^{\left(i\right)}$ and static features *c*^(*i*)^. The $x_{t}^{\left(i\right)}$ and $z_{t}^{\left(i\right)}$ are generated for each patient at a given time *t* through an autoregressive process, and these generation processes take into account as well as the influence of previous treatment assignments, so we define the following equations to generate covariates *x* and hidden confounders *z*,

16 $$x_{t,j}^{\left(i\right)}=\frac{1}{p}\sum_{r=1}^{p}\left(\alpha_{r,j}x_{t-r,j}^{\left(i\right)}+\beta_{r}a_{t-r}^{\left(i\right)}\right)+\eta_{t}\;z_{t,j}^{\left(i\right)}=\frac{1}{p}\sum_{r=1}^{p}\left(\mu_{r,j}z_{t-r,j}^{\left(i\right)}+v_{r}a_{t-r}^{\left(i\right)}\right)+\epsilon_{t}$$

where $x_{t,j}^{\left(i\right)}$ and $z_{t,j}^{\left(i\right)}$ denote the *j*-th column of $x_{t}^{\left(i\right)}$ and $z_{t}^{\left(i\right)}$, respectively. For each *j*, weuse*α*_*r*,*j*_, *μ*_*r,j*_ ~ 𝒩 (1 − (*r*/*p*), (1/*p*)^2^) to control the amount of historical information of last *p* time stamps incorporated to the current representations; *β*_*r*_, *v*_*r*_ ~ 𝒩 (0, 0.02^2^) controls the influence of previous treatment assignments; *η*_*t*_, *ϵ*_*t*_ ~ 𝒩 (0, 0.01^2^) are randomly sampled noises. The treatment assignments are generated by creating 1,000 treated samples and 3,000 control samples, with treatments starting at a randomly chosen point for treated samples and all treatments set to 0 for control samples. The confounders $q_{t}^{\left(i\right)}$ and outcome $y_{T+\tau }^{\left(i\right)}$ at each time stamp *t* are generated using the hidden confounders and current covariates as follows,

17 $$q_{t}^{\left(i\right)}=\gamma \frac{1}{t}\sum_{r=1}^{t}z_{r}^{\left(i\right)}+(1-\gamma )g\left(\left[x_{t}^{\left(i\right)},c^{\left(i\right)}\right]\right)\;y_{T+\tau }^{\left(i\right)}=w^{\backslash \text{top}}\;q_{T}^{\left(i\right)}+b$$

where the influence of hidden confounders being controlled by a confounding factor *γ*, and *w* ~ 𝒰 (−1,1) and b ~ 𝒩 (0,0.1) are weights and biases of a linear model. The function g(·)maps the concatenated feature vectors $\left[x_{t}^{\left(i\right)},c^{\left(i\right)}\right]$ into the hidden space. For this study, we used confounding factor *γ*=0.1, 100 covariates, and 10 time steps when generating the samples.

#### 3.1.2 The MIMIC-III dataset.

Following the similar setting of Bica et al [23], we constructed a dataset based the Medical Information Mart for Intensive Care version III (MIMIC-III) [35]. The MIMIC-III dataset contains more than 61,000 ICU admissions from 2001 to 2012 with recorded patients’ demographics and temporal information, including vital signs, lab tests, and treatment decisions. We extracted 11,715 adult sepsis patients fulfilling the sepsis3 criteria [36] as our studied cohort from MIMIC-III.

Here, we obtain 27 time-varying variables (i.e., vital signs: temperature, heart rate, systolic, mean blood pressure (MBP), diastolic blood pressure, respiratory rate, oxygen saturation (SpO2); lab tests: sodium, chloride, magnesium, glucose, blood urea nitrogen, creatinine, urineoutput, glasgow coma scale, white blood cells count, bands, C-Reactive protein, hemoglobin, hematocrit, aniongap, platelets count, partial thromboplastin time, prothrombin time, international normalized ratio, bicarbonate, lactate) and 8 static demographics (i.e., age, gender, race, metastatic cancer, diabetes, height, weight, body mass index) variables. We design two causal inference tasks considering two available treatment assignments: vasopressors and mechanical ventilator (MV). For each treatment option, we separately evaluate its causal effect on the important outcomes of interest. For vasopressors, we adopted MBP as the target outcome; and for mechanical ventilator, we adopted the SpO2 as the outcome. We consider the rest of the variables as the observed covariates.

#### 3.1.3 The NACC dataset

Follow a similar process, we construct the longitudinal data from the National Alzheimer’s Coordinating Center (NACC) Uniform Data Set (UDS) [37]. The NACC-UDS is a database that collects demographic, clinical, diagnostic, and neuropsychological data from 29 Alzheimer’s Disease Centers (ADCs) from recruited participants with normal cognition, mild cognitive impairment (MCI), and dementia at baseline and being followed annually, since 2005. We collected data from the NACC-UDS between June 2005 and June 2021 to formulate 2 separate datasets with patients of different baseline conditions, i.e., (1) baseline-1: patients who were diagnosed with MCI and age above 50; and (2) *baseline-2*: patients with normal cognition and age above 65. We extracted 2,401 and 5,555 patients for baseline-1 and baseline-2 respectively with over 268 variables, and the detailed variables’ information can be found in the Appendix A section. We considered three treatments assignments, i.e., statin, anti-hypertensive, and non-steroidal anti-inflammatory drugs (NSAID) and aim to estimate their effects on reducing the risk of Alzheimer’s disease (AD).

#### 3.1.4 Results

Table 1 demonstrates the superior performance of our VTD model in terms of both RMSE and IF-PEHE on the synthetic data. This highlights the ability of the VTD model’s variational embedding that can effectively capture the information of hidden confounders within a temporal structure, resulting in a more accurate estimation of ITE. Furthermore, the deep representation-based models exhibit a significant improvement over the baseline G-formula, attributed to their capability to handle complex and high-dimensional data through the utilization of neural networks as the underlying architecture.

Table 2 presents the evaluation of VTD’s effectiveness in deconfounding by assessing its performance with different strengths (i.e., adjusting γ) of hidden confounders Z. The setting is similar to the previous experiment on synthetic data, and we report the RMSE on the outcome prediction as the performance metric. The results indicate that the proposed VTD outperforms the other baselines and the performance of the VTD increases when the confounding factorγ increases. It should be noted that both baselines and VTD are evaluated on the same data, thus the performance gain is due to VTD’s more effective modeling of hidden confounders. The results demonstrate that conditioning on the hidden embedding learned by VTD results in more robust outcome predictions and reduces the bias in ITE estimation.

We evaluate the performance of the VTD on the benchmark MIMIC-III dataset which a real-world dataset. So we don’t have the knowledge of the true hidden confounders in this dataset. Table 3 demonstrates that the VTD model outperforms both the TSD and the G-formula on all measures and provides better outcome predictions in the “ vasopressor-MBP “ setting, with similar performance in the “ MV-SpO2” setting compared to DSW on the MIMIC-III dataset. This indicates that the VTD, with its time-aware Transformer backbone, can benefit from learning the patterns of irregular elapsed time between consecutive events.

Table 4 and Table 5 show the performance of four models on baseline-1 and baseline-2, respectively. We see VTD gains more edges on both settings for outcome prediction power. While we have did not observe better performance of IF-PEHE for VTD.

## 4 Discussion And Conclusion

In this paper, we introduced a novel approach, the Variational Temporal Deconfounder (VTD), for estimating the individual treatment effect (ITE) in a longitudinal setting. The method addresses the problem of hidden confounding, which is a critical issue in ITE estimation from observational data such as electronic health records (EHRs). We demonstrated the effectiveness of VTD’s deconfounding ability with synthetic data over different strengths of confounding factor. The results of the two experiments on synthetic data are consistent and demonstrate that the VTD consistently outperforms existing methods in terms of ITE estimation accuracy and IF-PEHE. In the real-world application using MIMIC-III, we can see VTD performs better than the baseline G-formula and the TSD model, similar to DSW on the IF-PEHE metric (with a few cases, where VTD performed worse than DSW). In the NACC dataset, we observe some superior results on outcome predictions and worse-but competitive-results on IF-PEHE comparing to DSW. However, DSW is a deep learning-based approach built on the assumption of unconfoundedness, which our VTD does not assume. It is also interesting that the VTD model performs well in the NACC dataset comparing to MIMIC-III, where the two real-world datasets capture different disease/application settings. The MIMIC-III data capture care in the ICU settings, while the NACC dataset captures the setting of chronic diseases (i.e., the development of AD). Thus, similar to the selection of traditional machine learning algorithms (e.g., support vector machine vs. random forest and others) for a prediction task that depends on the assumptions of the underlying data distributions, the selection of an appropriate ITE estimation methods really depend on our assumptions (or no assumption of) of the underlying causal structures (e.g., whether there exists hidden confounders and whether there are proxies exist for the hidden confounders), which explains some of the variations of the model performance across different datasets and settings.

The overall improvement of the VTD model lies in its ability to address the problem of hidden confounding in a longitudinal setting, which were not addressed in most previous ITE estimation methods. The use of auto-encoded variational inference allows the model to create latent variables that recover the distributions of hidden confounders, making it possible to estimate ITEs even in the presence of hidden confounders.

However, there are some limitations to the VTD model. First, the VTD model assumes proxies for hidden confounders are available in the observational data. In cases where these proxies are not available, the VTD may not be the most suitable choice. Second, our evaluation of the VTD in the real-world applications is limited to a surrogate metric IF-PEHE, while true gold-standards are not available.

In sum, the ability to estimate ITEs in a longitudinal setting while taking into account the existence of hidden confounders makes the VTD model particularly useful for personalized medicine, where the goal is to optimize treatment choices for individual patients based on their unique characteristics observed over time. Nevertheless, further investigations are needed as the unconfoundedness assumption may not hold in certain real-world applications. Identifying the types of real-world applications where unconfoundedness holds or not is thus critical to guide the choice of the modeling approach.

## Figures and Tables

**Table 1:** Performance comparison on the synthetic dataset in terms of RMSE and IF-PEHE.

Model	RMSE	IF-PEHE
G-formula	5.46 ±0.11	30.42 ± 4.64
DSW	2.63 ±0.05	10.28 ± 1.06
TSD	3.06 ± 0.14	23.65 ± 2.23
**VTD (Ours)**	**2.07 ± 0.12**	**8.31 ± 1.59**

**Table 2:** Performance comparison (i.e., RMSE) of models with different confounding factor γ on the synthetic data.

Model	γ = 0	γ = 0.2	γ = 0.4	γ = 0.6
**G-formula**	3.86 ± 0.12	7.41 ± 1.53	13.72 ± 3.29	16.43 ± 4.24
**DSW**	1.95 ± 0.05	3.28 ± 0.14	7.23 ± 0.35	9.17 ± 0.61
**TSD**	2.89 ±0.10	5.51 ± 0.21	9.79 ± 0.78	11.65 ± 1.54
**VTD (Ours)**	**1.78 ± 0.08**	**2.96 ± 0.16**	**4.16 ± 0.21**	**7.62 ± 0.69**

**Table 3:** Performance comparison on the MIMIC-III dataset.

Model	Vasopressor on Mean Blood Pressure (Vasopressor-MBP)		Mechanical Ventilator on SpO2 (MV-SpO2)	
	RMSE	IF-PEHE	RMSE	IF-PEHE
**G-formula**	12.53 ± 0.27	63.35 ± 5.43	1.57 ± 0.14	53.28 ± 5.21
**DSW**	8.55 ± 0.22	**12.01 ± 2.33**	**1.06 ± 0.11**	**8.68 ± 1.54**
**TSD**	9.34 ± 0.10	57.26 ± 4.71	1.23 ± 0.07	35.21 ± 4.85
**VTD (ours)**	**8.36 ± 0.14**	20.16 ± 2.10	1.12 ± 0.13	17.25 ± 1.78

**Table 4:** Model performance comparison on NACC dataset for baseline-1* setting.

Model	AD-Statin		AD-Anti-hypertensive		AD-NSAID	
	RMSE	IF-PEHE	RMSE	IF-PEHE	RMSE	IF-PEHE
**G-formula**	22.16 ± 1.24	102.11 ± 12.21	21.46 ± 0.98	95.87 ± 10.67	25.23 ± 1.27	96.89 ± 11.54
**DSW**	**9.43 ± 0.16**	**37.45 ± 2.12**	11.37 ± 0.11	**40.28 ± 3.27**	9.25 ± 0.13	**39.42 ± 3.54**
**TSD**	13.72 ± 0.18	73.43 ± 3.21	13.27 ± 0.17	85.27 ± 3.69	15.26 ± 0.15	65.43 ± 4.78
**VTD (ours)**	9.79 ± 0.15	42.43 ± 3.25	**9.42 ± 0.12**	58.86 ± 4.18	**7.43 ± 0.11**	46.59 ± 5.35

* baseline-1: patients who were diagnosed with mild cognitive impairment (MCI) and age above 50.

**AD**: Alzhemeri’s disease (AD)

**NSAID**: non-steroidal anti-inflammatory drug

**Table 5:** Model performance comparison on NACC dataset for baseline-2* setting.

Model	AD-Statin		AD-Anti-hypertensive		AD-NSAID	
	RMSE	IF-PEHE	RMSE	IF-PEHE	RMSE	IF-PEHE
**G-formula**	20.16 ± 1.19	84.28 ± 15.28	27.16 ± 1.35	89.29 ± 11.37	19.67 ± 1.14	99.17 ± 15.93
**DSW**	**7.28 ± 0.31**	**43.57 ± 2.15**	10.46 ± 0.19	**38.27 ± 2.57**	7.81 ± 0.16	**25.48 ± 2.18**
**TSD**	9.79 ± 0.15	64.35 ± 3.67	12.67 ± 0.21	74.28 ± 2.21	9.26 ± 0.13	59.37 ± 3.51
**VTD (ours)**	7.38 ± 0.24	37.25 ± 3.16	**10.39 ± 0.27**	40.74 ± 3.29	**6.98 ± 0.12**	32.52 ± 3.24

* baseline-2: patients with normal cognition and age above 65.

**AD**: Alzhemeri’s disease (AD)

**NSAID**: non-steroidal anti-inflammatory drug
